# The relationship between sleep problems and well-being among Chinese children: a moderated mediation model with anxiety as mediator and resilience as moderator

**DOI:** 10.3389/fpsyg.2026.1900442

**Published:** 2026-10-23

**Authors:** Junfeng Ding, Xinhua Hou, Guiping Dou

**Affiliations:** 1 School of Education and Humanities, Suzhou Polytechnic University, Suzhou, Jiangsu, China; 2 Inclusive Education and Children's Health Research Center, Suzhou Polytechnic University, Suzhou, Jiangsu, China; 3 Suzhou Hi-Tech Zone Fengqiao Guanfeng Kindergarten, Suzhou, Jiangsu, China; 4 School of Educational Sciences, Zhoukou Normal University, Zhoukou, Henan, China

**Keywords:** anxiety, moderated mediation analysis, resilience, sleep problems, well-being

## Abstract

**Objective:**

Prior research has shown that well-being is a key factor in children’s developmental outcomes. However, the factors that relate to children’s well-being are still unclear, especially among Chinese populations. This research investigated the relationship between sleep problems and the well-being of children in China, specifically testing (1) whether sleep problems were associated with well-being, (2) whether anxiety may serve as a mediator, and (3) whether resilience may moderate this association.

**Methods:**

A total of 606 Chinese children (53.3% girls; mean age = 11.09 years) participated in the present study using a cross-sectional design by completing self-report instruments, including the Athens Insomnia Scale, the Oxford Happiness Questionnaire, the Anxiety subscale of the Depression Anxiety Stress Scale, and the Connor-Davidson Resilience Scale. Pearson correlations and a moderated mediation analysis using PROCESS Model 58 with 5,000 bootstrap samples were conducted, controlling for age.

**Results:**

(1) There was a significant negative correlation between sleep problems and well-being (*r* = −0.39, *p* < 0.001). (2) After controlling for age, anxiety mediated this association. (3) Resilience moderated this association. Specifically, the indirect effect was stronger in the high-resilience group (indirect effect = −0.15, *95% CI* = [−0.23, −0.08]) than in the low-resilience group (indirect effect = −0.05, *95% CI* = [−0.09, −0.03]).

**Conclusion:**

These findings provide preliminary evidence on how sleep problems are associated with well-being among Chinese children. They also suggest that anxiety and resilience may be relevant for future intervention studies, although longitudinal and experimental designs are needed to test whether targeting these factors can improve mental health.

## 1 Introduction

As a core measure of mental health, well-being reflects a person’s ability to achieve self-actualization and lead a meaningful, purposeful life (Chen et al., 2013; Hsieh, 2016). A large body of evidence consistently ties children’s well-being to favorable life experiences, adaptive school functioning, and academic success (Altıntaş and Canbulat, 2024; Dempsey et al., 2023; Luo et al., 2023). In one illustrative study, Dempsey et al. (2023) demonstrated that well-being in children predicts their later altruistic behavior and academic self-concept. Collectively, such findings suggest that well-being meaningfully shapes a wide range of developmental outcomes. Thus, identifying the factors associated with well-being among Chinese children becomes a pressing question. Answers to this question could inform interventions and strategies that advance children’s psychological health.

### 1.1 Sleep problems and well-being

There were several studies on the factors associated with children’s well-being, such as emotional intelligence and school environments (Di Fabio and Kenny, 2016; Tapia-Fonllem et al., 2020). One crucial factor for the well-being of children is sleep. It has become a growing public concern, largely because of its key role in an individual’s psychological, cognitive, and behavioral functioning (Astill et al., 2012; Medic et al., 2017; Morales-Muñoz et al., 2021). Especially for children, sleep is one of the leading factors of psychological development and mental health (Schlieber and Han, 2021). Research has consistently highlighted a significant negative correlation between sleep problems and children’s well-being (Matricciani et al., 2019; Neubauer et al., 2021; Williamson et al., 2020). The study of Williamson et al. (2020) found that children with sleep problems had marked impairments in their well-being. Similarly, Thumann et al. (2019) showed that higher well-being was linked to longer sleep duration and fewer sleep disturbances. James and Hale (2017) documented a consistently nonlinear, U-shaped pattern in the sleep duration–well-being association among children. Given the above, we hypothesized a significant negative relationship between sleep problems and well-being among Chinese children.

### 1.2 The roles of anxiety and resilience

Moreover, existing literature suggests that the association between sleep problems and well-being is not straightforward, but rather complex and potentially shaped by various mediating and moderating factors (Su and He, 2023). These intervening variables may help explain individual differences in how sleep issues are associated with psychological outcomes. Guided by the Double ABCX model—a dominant analytical framework for stress and coping in family systems—well-being (as a mental health outcome) is considered to be shaped by the dynamic interplay between several key elements: sleep problems as the initial stressor, anxiety as the individual’s cognitive-emotional appraisal of that stressor, and resilience as a personal resource in the stress-coping process (McCubbin and Patterson, 1983). This model provides a valuable lens through which to investigate how different psychological processes may jointly relate to children’s well-being in the context of sleep problems.

Childhood anxiety, conceptualized as a persistent negative emotional response to perceived threats or stressors (Ding et al., 2023), when present at clinically relevant levels, frequently is associated with diverse psycho-behavioral impairments (Liang et al., 2021; Mulud et al., 2019). Neurobiological research has elucidated the complex interplay between sleep regulation mechanisms and anxiety pathways, revealing robust bidirectional connections that persist across different age cohorts (Chellappa and Aeschbach, 2022; Narmandakh et al., 2020; Shimizu et al., 2021). Especially for children and early adolescents, sleep problems may be linked to heightened anxiety (Narmandakh et al., 2020). Additionally, A statistically significant link between anxiety and well-being emerged in children and adolescents (Jiang et al., 2018; Malone and Wachholtz, 2018). Existing research has consistently demonstrated that anxiety functions as a critical mediator in the association between adverse life events and diminished well-being (Jiang et al., 2018). More importantly, anxiety mediated the relationship between the quality of sleep and well-being among Chinese adults (Li X. R. et al., 2022). Therefore, anxiety likely played a mediating role in linking sleep problems with well-being in Chinese children.

Recent clinical work has linked poorer sleep quality to lower psychological resilience and lower mental well-being (Aksakal et al., 2025), and similar associations have been observed in young children (Ding et al., 2023). These findings suggest that resilience may be an important psychological factor in the sleep–well-being relationship. Given its potential role in this relationship, resilience has been examined as a moderator of the association between environmental stressors and psychological outcomes (Jiang et al., 2020). This study adopts the contemporary definition of resilience as the capacity to maintain or restore psychological homeostasis when facing major life disruptions and prolonged adversity (American Psychological Association, 2020). A growing body of literature suggests the dual nature of resilience, showing negative associations with psychopathological outcomes while being positively associated with well-being and positive affectivity (Jiang et al., 2020; Wang and Hong, 2023). Higher resilience is associated with lower anxiety (Ding et al., 2023), and resilience has also been linked to better subjective well-being among Chinese left-behind children (Zhou et al., 2021). Additionally, previous studies found a moderating role of resilience in the relationship between negative experiences (e.g., bullying) and anxiety (Wu et al., 2018). Similar moderating effects of resilience have been found between negative experiences and well-being among Chinese adolescents (Jiang et al., 2020; Zhang et al., 2024). Although these studies were conducted in different populations (e.g., adolescents, bullying contexts), they provide preliminary evidence for the moderating role of resilience. Therefore, we hypothesized that resilience would moderate the indirect association between sleep problems and well-being via anxiety in Chinese children.

The present study examined the relationship between sleep problems and well-being and the potential indirect pathway underlying this relationship in Chinese children (See **Figure** **1**). This study proposed three key research hypotheses: (1) A significant negative association would exist between sleep problems and well-being in Chinese children; (2) Anxiety would serve as a significant mediating variable in the sleep problems-well-being relationship; (3) Resilience moderated the link between sleep problems and well-being.

**Figure 1:**
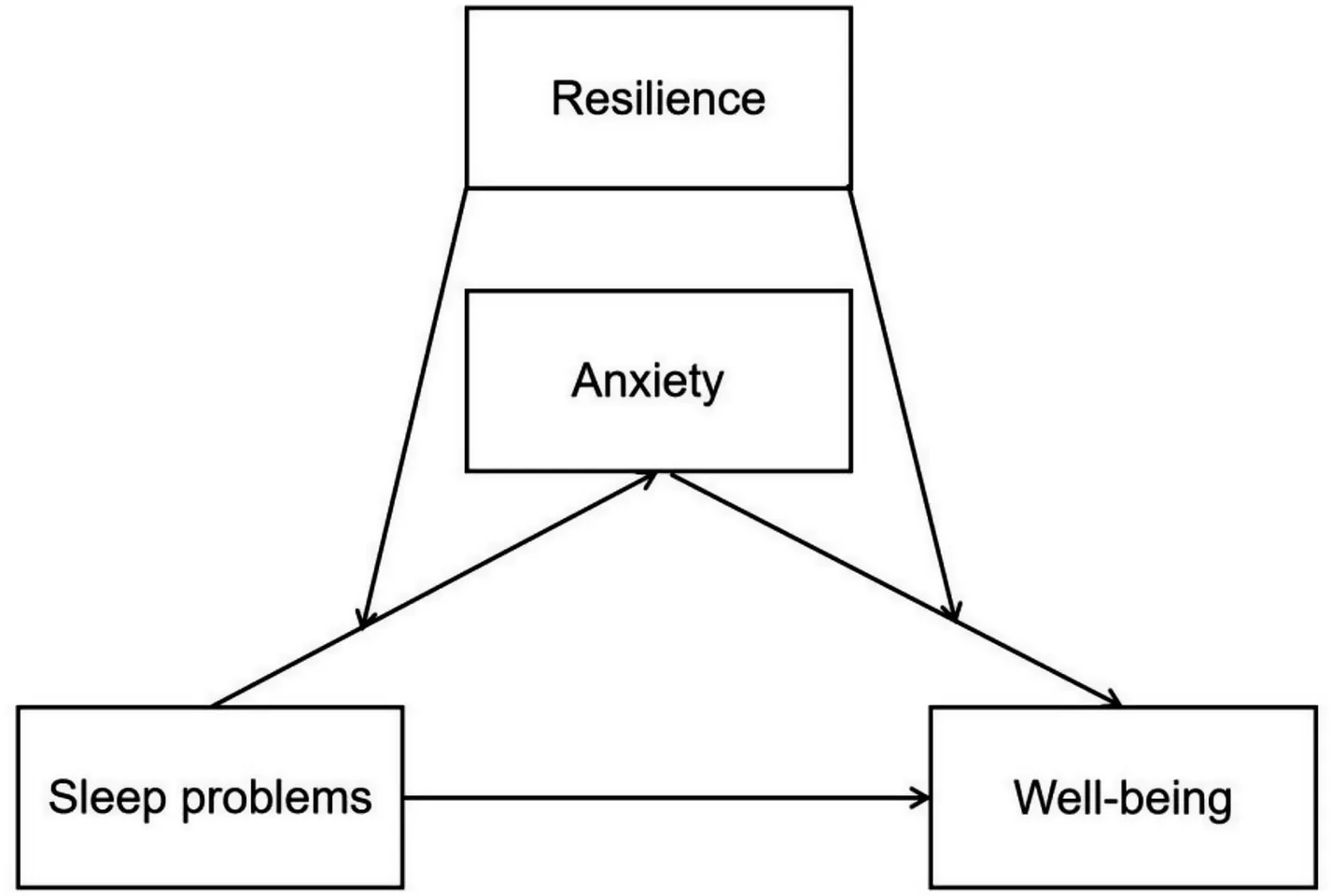
The theoretical moderated mediation model.

## 2 Methods

### 2.1 Participants

Participants were recruited from two public primary schools in Guizhou Province, China, using a cluster sampling method. Data were collected between September and October 2024. This study obtained ethical approval from the School of Educational Sciences, Zhoukou Normal University, China (Approval No. ZKU-JYXY-202405). Informed consent was obtained from the legal guardian(s) of all subjects before the investigation, and the study will not cause any harm to the child or their family. Personal information of the children will be kept strictly confidential unless required by law.

Inclusion criteria were: (a) age 9–12 years, (b) able to read and understand Chinese, (c) no diagnosed psychiatric disorders, and (d) provided complete responses to the required self-report measures. The study sample initially comprised 667 Chinese children aged 9–12 years, of whom 61 participants (representing 9.14% of the total sample) were excluded during data screening procedures. This exclusion criterion was applied based on incomplete responses to the required self-report measures. The final analytical sample comprised 606 children aged 9–12 years (*M*_age_ = 11.09 years, *SD*_age_ = 1.00). Approximately 46.7% of the children were boys, and 53.3% were girls.

### 2.2 Measures

#### 2.2.1 The Athens insomnia scale

The sleep problems experienced by Chinese children were assessed quantitatively using the Athens Insomnia Scale (AIS), which was initially developed by Soldatos et al. (2000). Although the AIS was originally developed for adults, the Chinese version has demonstrated acceptable reliability and validity in Chinese adolescents (Lu et al., 2024). This scale includes 8 items, each rated on a four-point Likert scale, with higher scores indicating more severe sleep problems. In the present study, the scale exhibited satisfactory internal consistency (Cronbach’s α = 0.79).

#### 2.2.2 The Oxford happiness questionnaire

The well-being of children was assessed using the 8-item version of the Oxford Happiness Questionnaire (OHQ), created by Hills and Argyle (2002). This questionnaire was completed using a six-point Likert scale, ranging from 1 (“strongly disagree”) to 6 (“strongly agree”). Higher scores reflect a greater level of well-being. In this study, Cronbach’s α was 0.66. This value is comparable to previous reports using the short form (Bağatarhan, 2025; Rubio-Belmonte and García-Alandete, 2025).

#### 2.2.3 The depression anxiety stress scale

Anxiety was assessed using the 7-item anxiety subscale from the Depression Anxiety Stress Scale (DASS-21), which was initially developed by Lovibond and Lovibond (1995). Prior research has demonstrated the scale’s strong psychometric properties among Chinese school-aged populations (Zhang et al., 2016). Each item is rated on a 4-point Likert scale assessing symptom frequency over the past week (0 = did not apply to me at all, 1 = applied to me to some degree or some of the time, 2 = applied to me to a considerable degree or a good part of the time, 3 = applied to me very much, or most of the time). The anxiety subscale score was calculated by averaging the seven items. Higher scores indicate greater levels of anxiety. In this study, the Cronbach’s α coefficient for the scale was 0.73.

#### 2.2.4 The 25-item Connor-Davidson resilience scale

Resilience was assessed using the 25-item Connor-Davidson Resilience Scale (CD-RISC), which utilizes a 5-point Likert scale (0 = “not true at all” to 4 = “true nearly all the time”) for responses (Connor and Davidson, 2003). The Chinese version of the CD-RISC has demonstrated good reliability and validity among Chinese adolescents (Fu et al., 2014). The resilience score was calculated by averaging the responses to all items on the scale, with higher scores reflecting stronger resilience. In this study, Cronbach’s α for the scale was 0.92.

### 2.3 Data analysis

All statistical procedures were carried out using IBM SPSS Statistics (Version 27.0). Before engaging in hypothesis testing, this study took steps to address methodological validity by employing Harman’s single-factor test to evaluate the presence of common method variance (Aguirre-Urreta and Hu, 2019). The results from an exploratory factor analysis indicated that the first unrotated principal component accounted for only 15.84% of the total variance, which is well below the threshold of 40%. This finding suggests that common method bias was unlikely to pose a significant threat to the integrity of our data. Subsequent analytical procedures involved three key steps: First, we explored potential gender differences in children’s well-being indicators by applying independent samples *t*-tests. Next, we calculated Pearson correlation coefficients to examine bivariate associations among all key variables. Finally, we employed Hayes’ PROCESS macro to investigate the hypothesized mediation and moderation effects (Hayes, 2017). To prepare the data for model estimation, all continuous predictor variables were standardized using z-score transformation. Outcome variables were analyzed on their original scales; therefore, the reported coefficients represent the change in the outcome associated with a one-standard-deviation increase in the predictor. This approach facilitated clearer interpretation and helped reduce issues of multicollinearity. The moderated mediation model was analyzed using PROCESS Model 58, based on 5,000 bias-corrected bootstrap samples. This bootstrapping method generated 95% confidence intervals for both direct and indirect effects, offering enhanced statistical precision and power relative to more traditional parametric approaches, particularly given the size of our sample.

## 3 Results

### 3.1 Descriptive statistics and correlation analyses

The First, the analysis indicated that gender did not have a statistically significant impact on well-being levels, *t_(604)_* = 0.90, *p* = 0.370. Given that examining gender differences was not a central goal of the present study, this variable was excluded from further analysis and discussion. Second, Table 1 contains the summary statistics for all variables and the corresponding correlation matrix based on Pearson’s coefficient calculations. The results found that sleep problems had a positive correlation with anxiety (*r* = 0.43, *p* < 0.001) and negative correlations with resilience (*r* = −0.35, *p* < 0.001) and well-being (*r* = −0.39, *p* < 0.001). Well-being had a positive correlation with resilience (*r* = 0.42, *p* < 0.001) and a negative correlation with anxiety (*r* = −0.41, *p* < 0.001). Anxiety had a negative correlation with resilience (*r* = −0.30, *p* < 0.001). Age had significant correlations with well-being (*r* = 0.13, *p* = 0.001) and anxiety (*r* = −0.11, *p* = 0.007), but did not have significant correlations with sleep problems and resilience (|*r*|s < 0.04, *p*s > 0.320).

**Table 1:** Descriptive statistics and Pearson correlations matrix (*n* = 606).

Variables	M	SD	1	2	3	4	5
1. Sleep problems	4.34	3.72	—				
2. Well-being	4.60	0.77	−0.39^***^	—			
3. Anxiety	1.64	0.59	0.43^***^	−0.41^***^	—		
4. Resilience	2.29	0.77	−0.35^***^	0.42^***^	−0.30^***^	—	
5. Age	11.09	1.00	−0.06	0.13^**^	−0.11^**^	0.04	—

M, mean, SD, standard deviation, ^*^p < 0.05, ^**^p < 0.01, ^***^p < 0.001.

### 3.2 The moderated mediation analyses

To examine the hypothesized mediation effect of anxiety and the moderating influence of resilience on the association between sleep problems and well-being, we conducted a series of regression analyses while controlling for participants’ age as a covariate (see Table 2). The analytical framework followed a two-stage approach to ensure methodological rigor. In equation 1, we found that sleep problems (*β* = 0.46, 95% *CI* [0.37, 0.55], *p* < 0.001) and resilience (*β* = −0.12, 95% *CI* = [−0.21, −0.03], *p* = 0.009) had direct effects on anxiety. Furthermore, the interaction of sleep problems and resilience significantly predicted anxiety (*β* = 0.13, 95% *CI* = [0.06, 0.21], *p* = 0.001). In equation 2, the results demonstrated significant predictive relationships between the independent variables and well-being. Specifically, sleep problems exhibited a negative association (*β* = −0.16, 95% *CI =* [−0.24, −0.09], *p* < 0.001). Similarly, anxiety showed an inverse relationship (*β* = −0.20, 95% *CI* = [−0.26, −0.14], *p* < 0.001), while resilience emerged as a positive predictor (*β* = 0.26, 95% *CI* = [0.20, 0.33], *p* < 0.001). Moreover, the interaction of anxiety and resilience significantly predicted well-being (*β* = −0.05, 95% *CI* = [−0.10, −0.01], *p* = 0.029). All 95% confidence intervals excluded zero, indicating a significant association between sleep problems and well-being, with anxiety acting as a mediator and resilience as a moderator.

**Table 2:** Moderated Mediation Analysis of Anxiety and Resilience on the Association Between Sleep Problems and Well-being in Chinese Children (*n* = 606).

Predictors	Outcomes	R^2^/ ΔR^2^	F	β	t	95% CI	
						LLCI	ULCI
Equation 1							
Sleep problems	Anxiety	0.23	45.32^***^	0.46	10.13^***^	0.37	0.55
Resilience				−0.12	−2.64^**^	−0.21	−0.03
Sleep problems × Resilience		0.02	11.94^***^	0.13	3.46^**^	0.06	0.21
Age				−0.07	−1.92	−0.15	0.00
Equation 2							
Sleep problems	Well-being	0.30	52.42^***^	−0.16	−4.48^***^	−0.24	−0.09
Anxiety				−0.20	−6.33^***^	−0.26	−0.14
Resilience				0.26	7.77^***^	0.20	0.33
Anxiety × Resilience		0.01	4.77^*^	−0.05	−2.18^*^	−0.10	−0.01
Age				0.07	2.18^*^	0.01	0.12

^*^p < 0.05, ^**^p < 0.01, ^***^p < 0.001. β represents the change in the outcome associated with a one-standard-deviation increase in the predictor.

Further, we explored the moderated role of resilience after controlling the effect of age. According to Hayes (2017), we transformed the standardized resilience into high level (≥ *M* + 1*SD*) and low level (≤ *M*-1*SD*). As shown in Table 2, resilience significantly moderated two paths: sleep problems → anxiety and anxiety → well-being. In Figure 2 and Table 3, we presented the moderating role of resilience in the relationship between sleep problems and anxiety. Analysis of the moderation effects revealed a differential pattern in the sleep-anxiety association contingent upon resilience levels. Children exhibiting greater resilience demonstrated a more pronounced positive relationship between sleep problems and anxiety (*β* = 0.60, 95% *CI* = [0.46, 0.73], *p* < 0.001) compared to their peers with lower resilience scores (*β* = 0.34, 95% *CI* = [0.24, 0.44], *p* < 0.001), suggesting resilience may amplify this psychopathological linkage. In Figure 3 and Table 3, we presented the moderating role of resilience in the relationship between anxiety and well-being. It was found that children with high levels of resilience had higher levels of well-being than children with low levels. The moderation analysis further indicated that resilience significantly influenced the strength of the anxiety-well-being association. Notably, children demonstrating higher resilience showed a more substantial negative coefficient (*β* = −0.25, 95% *CI* = [−0.33, −0.17], *p* < 0.001) compared to those with lower resilience levels (*β* = −0.15, 95% *CI* = [−0.23, −0.08], *p* < 0.001). This pattern suggests that heightened resilience may exacerbate the detrimental impact of anxiety on well-being.

**Figure 2:**
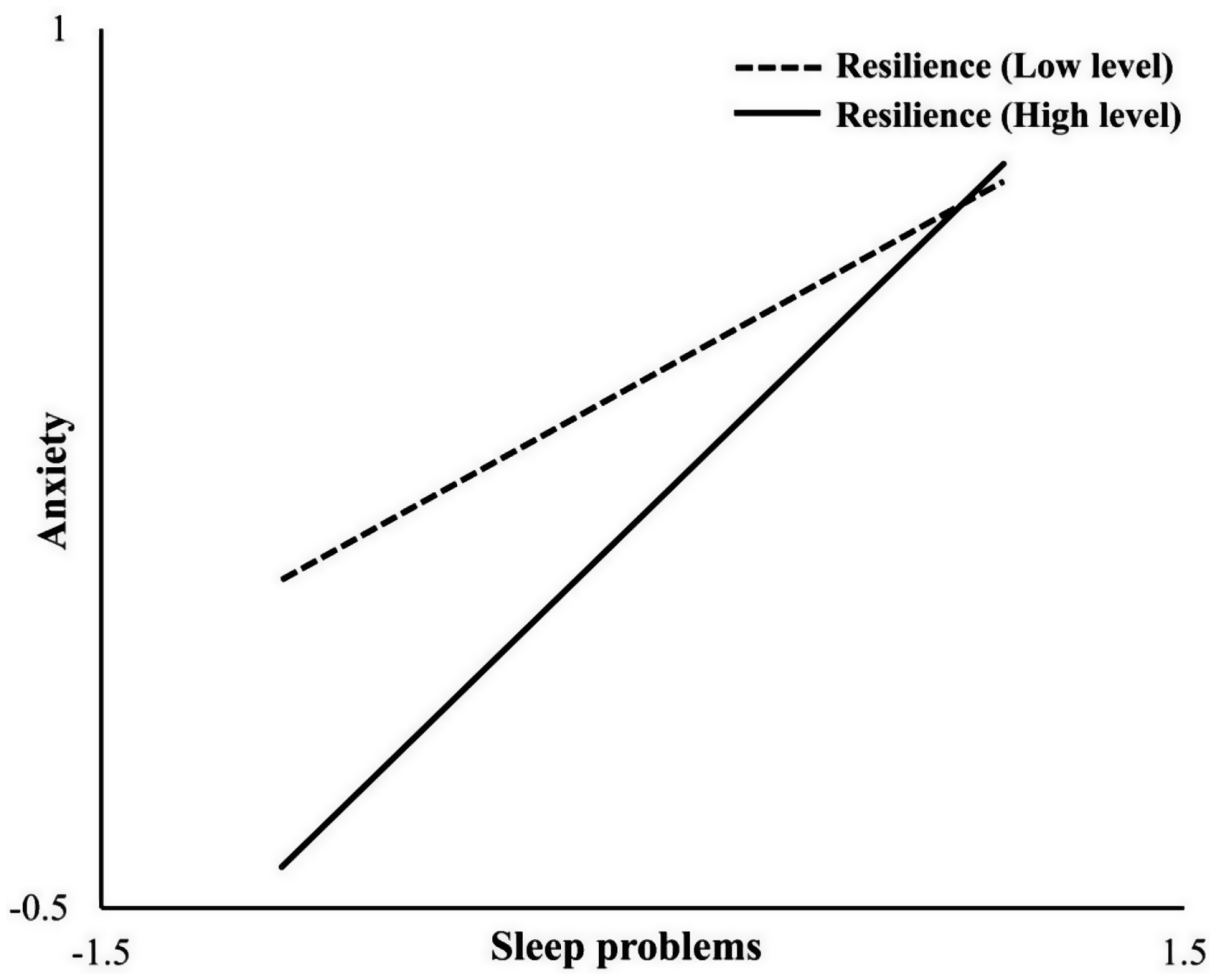
The moderating effect of resilience on the relationship between sleep problems and anxiety (*n* = 606). Low resilience = mean −1 *SD*; high resilience = mean +1 *SD*.

**Table 3:** Conditional associations of sleep problems on anxiety and anxiety on well-being at low and high levels of resilience (*n* = 606).

Path	Resilience	*β*	SE	*t*	95% CI	
					LLCI	ULCI
Sleep problems→Anxiety	Low level	0.34	0.05	6.93***	0.24	0.44
	High level	0.60	0.07	8.76***	0.46	0.73
Anxiety→Well-being	Low level	−0.15	0.04	−4.10***	−0.23	−0.08
	High level	−0.25	0.04	−6.18***	−0.33	−0.17

^***^p < 0.001. Low resilience = mean − 1 SD; high resilience = mean + 1 SD. β represents the change in the outcome associated with a one-standard-deviation increase in the predictor.

**Figure 3:**
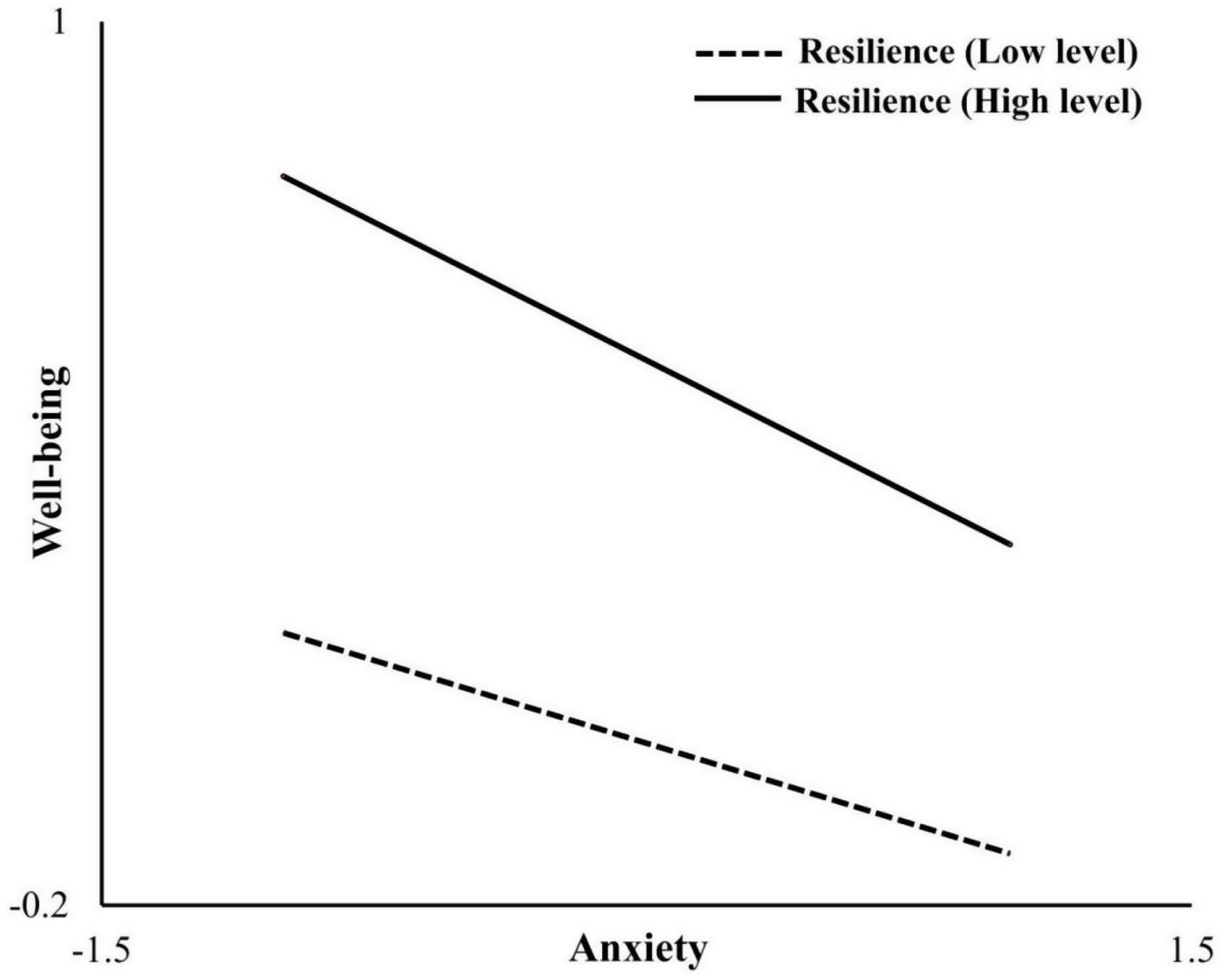
The moderating effect of resilience on the relationship between anxiety and well-being (*n* = 606). Low resilience = mean −1 *SD*; high resilience = mean +1 *SD*.

The moderated mediation results (Table 4) presented the direct and indirect effect sizes. These findings demonstrated that the total indirect effects were −0.05 (95% *CI =* [−0.09, −0.03]) and −0.15 (95% *CI =* [−0.23, −0.08]) for children with low and high levels of resilience, which accounted for 23.8 and 48.4% of the total effect.

**Table 4:** Direct and indirect effects of sleep problems on well-being (*n* = 606).

Effect		Total effect		Direct effect	Indirect effects	
		Resilience low level	Resilience high level		Resilience low level	Resilience high level
Effect estimate		−0.21	−0.31	−0.16	−0.05	−0.15
Proportion of total effect (%)		—	—	—	23.8%	48.4%
95% CI	LLCI	—	—	−0.24	−0.09	−0.23
	ULCI	—	—	−0.09	−0.03	−0.08

## 4 Discussion

This study utilized empirical approaches to explore the intricate relationship between sleep problems and well-being in Chinese children, with particular focus on the mediating role of anxiety and the moderating role of resilience. The findings support the hypotheses, highlighting the dynamic interactions between sleep issues, well-being, anxiety, and resilience within a sample of Chinese children. These results offer important insights into the connection between sleep disturbances and well-being, as well as the factors that shape this relationship in pediatric populations in China.

### 4.1 Sleep problems and well-being

Consistent with previous research, our results confirmed that sleep problems were robustly negatively associated with children’s well-being (Schlieber and Han, 2021; Williamson et al., 2020). As sleep problems increased, children reported lower levels of well-being. For children, sleep problems may be associated with poor physical health (e.g., obesity), cognitive functioning impairment, behavioral problems (e.g., internalizing problems), and social–emotional functioning impairment, which have negative implications for their overall well-being (Schlieber and Han, 2021). The documented prevalence of sleep problems among Chinese children, estimated at approximately 64% based on epidemiological studies, coupled with the established association between sleep quality and psychological well-being, underscores the urgent need for evidence-based interventions (Li X. L. et al., 2022).

### 4.2 The roles of anxiety and resilience

This study contributes to the limited body of research investigating the potential processes underlying the relationship between sleep problems and well-being among Chinese children. Using the Double ABCX model as a framework, the study explored the roles of anxiety and resilience within a moderated mediation model. Path analysis revealed both direct and indirect effects of sleep problems on well-being, with anxiety acting as a mediator. Specifically, greater sleep problems were associated with higher anxiety, which, in turn, was associated with poorer well-being. Previous research has identified sleep disturbances (e.g., insomnia) as a significant predictor of anxiety (Chellappa and Aeschbach, 2022; Hertenstein et al., 2019). Children with severe sleep issues may not only worry about insufficient sleep but also the potential consequences, such as impaired academic performance, maladaptive behaviors, and physiological hyper-arousal (Chellappa and Aeschbach, 2022; Williamson et al., 2020). As a result, sleep problems can exacerbate or perpetuate anxiety. Building on established theoretical frameworks, the current findings highlight anxiety as a critical mediator connecting sleep disturbances to diminished well-being in pediatric populations. The potential mediating role aligns with existing empirical studies that emphasize anxiety’s central role in linking negative developmental experiences to adverse mental health outcomes (Jiang et al., 2018).

The analyses further indicated that resilience moderated the indirect association between sleep problems and well-being via anxiety, and that this indirect association differed significantly across resilience levels. Specifically, the positive relationship between sleep problems and anxiety was stronger among children with higher resilience than among those with lower resilience. This suggests that the association between sleep problems and anxiety may be more pronounced at higher levels of resilience. This result aligns with prior research indicating that the association between adverse experiences and negative affect tends to be more pronounced in individuals with higher resilience capacities (Annor and Amponsah-Tawiah, 2020; Zhang et al., 2024). One possibility, which remains speculative and requires further investigation, is that children with higher resilience may be more aware of or more responsive to sleep-related difficulties. This is partly consistent with the “dark side of resilience” perspective, which suggests that higher resilience does not always confer protection and may sometimes amplify distress (Zhang et al., 2024).

With respect to the association between anxiety and well-being, children with higher resilience reported greater well-being overall and also showed a stronger negative association between anxiety and well-being than those with lower resilience. Thus, although higher resilience was associated with better well-being overall, the negative association between anxiety and well-being was stronger, not weaker, at higher levels of resilience. This pattern does not appear to support a straightforward buffering effect of resilience. One possibility, consistent with recent findings in other adversity contexts, is that individuals with higher resilience may expend considerable effort trying to manage persistent difficulties, and this overinvestment may deplete limited coping resources and strengthen the association (Zhang et al., 2024). Given the cross-sectional design, this interpretation remains speculative.

Children with higher resilience may be better able to manage sleep-related stress. Consequently, resilience may play a more complex role in the sleep–well-being association. Higher resilience was associated with better well-being overall, but the moderating pattern observed here suggests that the role of resilience may not be a simple buffering effect. These findings suggest that resilience and anxiety may be relevant for future intervention studies, although longitudinal and experimental designs are needed to test whether targeting these factors can improve children’s mental health.

## 5 Implications and limitations

The present study advances current knowledge in two important domains. Theoretically, our results not only corroborate existing evidence regarding the association between sleep problems and diminished well-being in pediatric populations, but more importantly elucidate the potential roles of anxiety and resilience in this relationship. These findings contribute to a more nuanced understanding of the potential processes linking sleep quality to psychological outcomes in children. From an applied perspective, the study offers empirically-grounded recommendations for developing integrated intervention protocols that address both the physiological and psychological dimensions of sleep-related difficulties.

Based on these findings, it is evident that future intervention studies targeting sleep disturbances should adopt a multifaceted approach that goes beyond merely addressing sleep problems. Specifically, interventions should incorporate psychological strategies that focus on anxiety reduction and resilience enhancement. When improvements in sleep quality alone are difficult to achieve, complementary strategies that target these psychological factors may help alleviate the negative impact of sleep problems on well-being. These insights are valuable for educators, clinicians, and mental health professionals involved in developing holistic, integrative intervention programs for children facing sleep-related challenges. By considering both the physiological and psychological aspects of sleep issues, practitioners can create more effective, comprehensive strategies to support children’s overall mental health and well-being.

Several methodological constraints warrant consideration when interpreting these findings. Most notably, the non-experimental, cross-sectional design precludes definitive causal inferences regarding the observed relationships. In addition, all main variables were assessed via child self-report, which may introduce common method bias, and objective or caregiver-reported measures of sleep were not included. Potential confounders such as socioeconomic status, health conditions, screen time, family functioning, and school characteristics were also not assessed. Regarding covariates, age was included based on prior evidence, whereas gender was excluded because it was not significantly associated with well-being; however, selecting covariates solely on univariable significance has limitations. The internal consistency of the Oxford Happiness Questionnaire was relatively low, which may have attenuated the observed associations involving well-being and should be considered when interpreting the findings. Furthermore, while the study examined anxiety as a mediator and resilience as a moderator, future longitudinal research should investigate additional biopsychosocial factors that may influence the sleep-wellbeing association. Finally, the sample was recruited from two public primary schools in Guizhou Province, which may limit generalizability. Such investigations would provide a more in-depth understanding of the complex interactions between sleep and psychological health in developing populations, and longitudinal and experimental studies are needed to clarify the role of resilience and to test whether targeting anxiety or resilience can improve children’s mental health.

## 6 Conclusion

This research systematically examined the underlying potential process involved in the association between sleep problems and well-being among Chinese children. This study provides preliminary evidence for a potential model in which anxiety mediates, and resilience moderates the sleep-well-being connection. These findings enrich theoretical accounts of the association between sleep problems and mental health and, at a practical level, may inform future research on Chinese children. However, given the cross-sectional design, these findings require confirmation through longitudinal and experimental studies.

## Data Availability

The raw data supporting the conclusions of this article will be available from the corresponding author upon request.
